# Absence of Vagina and Reconstruction

**Published:** 1913-04

**Authors:** 


Absence of Vagina and Reconstruction. Ernest F. Tucker of Portland, Ore., *N. W. Medicine*, January, 1913. The girl, aged 22, had been born with an imperforate anus operated upon immediately. On operation, a rudimentary uterus with normal right tube and ovary were found, accounting for the menstrual molimina. The vagina was successfully constructed from a loop of ileum.

